# Therapeutic management of traumatic facial palsy: a systematic review

**DOI:** 10.1007/s00405-025-09367-z

**Published:** 2025-04-26

**Authors:** Alice Ottavi, Anna Cozzi, Fabiana Allevi, Christian Calvo-Henriquez, Carlos Chiesa-Estomba, Giovanni Felisati, Jerome R. Lechien, Antonino Maniaci, Miguel Mayo-Yáñez, Giancarlo Pecorari, Giuseppe Riva, Luigi Angelo Vaira, Alberto Maria Saibene, Anastasia Urbanelli

**Affiliations:** 1 Otorhinolaryngology Unit, Department of Health Sciences, Santi Paolo e Carlo Hospital, University of Milan, Milan, Italy; 2 Maxillofacial Surgery Unit, Department of Health Sciences, Santi Paolo e Carlo Hospital, Università degli Studi di Milano, Milan, Italy; 3 Service of Otolaryngology, Hospital Complex of Santiago de Compostela, Santiago de Compostela, Spain; 4 Department of Otolaryngology-Head and Neck Surgery, San Sebastian University Hospital, San Sebastian, Spain; 5 Department of Otorhinolaryngology and Head and Neck Surgery, CHU de Bruxelles, CHU Saint-Pierre, School of Medicine, Brussels, Belgium; 6 Faculty of Medicine and Surgery, University of Enna “Kore”, Enna, Italy; 7 Otorhinolaryngology - Head and Neck Surgery Department, Complexo Hospitalario Universitario A Coruña (CHUAC), A Coruña, Galicia, 15006 Spain; 8 Otorhinolaryngology Unit, Department of Surgical Sciences, University of Turin, Turin, Italy; 9 Maxillofacial Surgery Operative Unit, Department of Medicine, Surgery and Pharmacy, University of Sassari, Sassari, Italy; 10 Department of Surgical Sciences, University of Turin, via G. Verdi, 8, Turin, 10124 Italy

**Keywords:** Traumatic facial palsy, Facial nerve, Facial palsy, Facial paralysis, Facial nerve injury

## Abstract

**Purpose:**

Trauma is a common cause of facial nerve palsy, accounting for 3% of all cases. While many facial palsies resolve with medical treatment, some require surgical intervention. This systematic review aimed to determine the best therapeutic strategy for traumatic facial palsy.

**Methods:**

We reviewed eligible articles for patient demographics, pre-treatment assessment, parameters of selected treatment, type of treatment, outcomes, and post-treatment assessment.

**Results:**

Among 135 unique citations, 32 studies were considered eligible, reporting treatment data for 2079 patients. Most studies (*n* = 30) were case series. The main proposed therapeutic strategies were medical, surgical, or a combination of both. For almost all the selected studies, the House-Brackmann (HB) scale was used to estimate the severity of facial palsy.

**Conclusion:**

Based on the existing literature, a standardized guideline for the treatment of traumatic facial palsy is not well delineated, due to the extreme heterogeneity of available therapeutic choices and the lack of standardized patient stratification.

## Introduction

Trauma to the facial nerve is one of the most frequent causes for facial nerve palsies (3% of all etiologies of facial palsy) [1]. While most facial palsies resolve by themselves or with medical treatment in weeks or months, some cases require a surgical approach. For example, early nerve decompression is indicated in acute complete traumatic palsies with 95% damage on electroneurography (ENoG) [2]. On the other hand, it is generally accepted that incomplete paralysis is more suitable for a “wait and see” approach.

The choice of the approach – conservative, medical, or surgical - has not yet been fully standardized. The indication for surgery varies between institutions, and there is no consensus on the optimal timing for surgical decompression or which cases would benefit most from surgical treatment [3, 4]. In the literature, many studies and reviews attempt to establish a common therapeutic pathway, though there is more agreement on how to classify the severity of facial palsy than on how to treat it. For example, computed tomography (CT) images, electroneuronography (ENoG), House–Brackmann (HB) grade, electromyography (EMG), audiological tests are all well-known ways for stratifying facial nerve injuries [5]. On the other hand, it seems more challenging to determine when and how to start therapy for each degree of severity and trauma. It is widely accepted that in cases of acute and complete traumatic facial palsy, with complete hearing loss, patients should undergo surgical treatment [5–7]. There are various surgical approaches to treating facial palsy, whether traumatic or not, and this review examines the most used techniques.

The purpose of this systematic literature review was to analyze the existing literature on traumatic facial palsy in order to propose guidelines for its management. Specifically, we analyzed pre-treatment assessment, type of treatment (conservative, surgery, or both), parameters for selecting the necessary treatment, the degree of deficit before treatment, and outcomes. Drawing on different experiences and decision-making processes, our aim is to clarify such a debated topic, considering the differences between types and degrees of trauma, and to identify tailored solutions based on the various existing studies.

## Materials and methods

### Search strategy

After registering with the Open Science Framework (OSF) database, we conducted a systematic review between July 17, 2023, and August 31, 2023, according to PRISMA reporting guidelines [8]. Systematic electronic searches were carried out in English, Italian, German, French, and Spanish, for articles reporting original data on traumatic facial palsies.

On July 17, 2023, a primary search was performed on the MEDLINE, Embase, Web of Science, Cochrane Library, Scopus, and ClinicalTrials.gov databases combining the terms “traumatic facial palsy” OR “traumatic facial paralysis”. Complete search strategies and the number of items retrieved from each database are provided in Table 1. The references of selected publications were then examined to identify further reports that were not found by database searching, and the same selection criteria were applied.

**Table 1:** Search strategy details and items retrieved from each consulted database

Database	Search date	Query	Items retrieved (*n*)
Medline	July, the 17th, 2023	(“traumatic facial palsy“[All Fields] OR “traumatic facial paralysis“[All Fields]) AND (“temporal fracture“[All Fields] OR “temporal bone fracture“[All Fields] OR “petrous fracture“[All Fields] OR “petrous bone fracture“[All Fields] OR “temporal trauma“[All Fields] OR “temporal bone trauma“[All Fields] OR (“petrous“[All Fields] AND (“injuries“[MeSH Subheading] OR “injuries“[All Fields] OR “trauma“[All Fields] OR “wounds and injuries“[MeSH Terms] OR (“wounds“[All Fields] AND “injuries“[All Fields]) OR “wounds and injuries“[All Fields] OR “trauma s“[All Fields] OR “traumas“[All Fields])) OR ((“petrous bone“[MeSH Terms] OR (“petrous“[All Fields] AND “bone“[All Fields]) OR “petrous bone“[All Fields]) AND (“injuries“[MeSH Subheading] OR “injuries“[All Fields] OR “trauma“[All Fields] OR “wounds and injuries“[MeSH Terms] OR (“wounds“[All Fields] AND “injuries“[All Fields]) OR “wounds and injuries“[All Fields] OR “trauma s“[All Fields] OR “traumas“[All Fields])))	154
Embase	July, the 17th, 2023	‘traumatic facial palsy’ OR ‘traumatic facial paralysis’	177
Cochrane library	July, the 17th, 2023	“traumatic facial palsy” OR “traumatic facial paralysis” in Title Abstract Keyword – (Word variations have been searched)	1
Web Of Science	July, the 17th, 2023	“traumatic facial palsy” OR “traumatic facial paralysis” (all fields)	50
Clinicaltrials.gov	July, the 17th, 2023	“traumatic facial palsy” OR “traumatic facial paralysis”	8
Scopus	July, the 17th, 2023	TITLE-ABS-KEY “traumatic facial palsy” OR “traumatic facial paralysis”	207
**Total non unique hits**			**597**

We included all article types excluding case reports, meta-analyses, and systematic or narrative reviews, which were nevertheless hand-checked for additional potentially relevant papers. Exclusion criteria were as follows: non-human studies, papers carried out in other languages than English, Italian, German, French, or Spanish, patients presenting non-traumatic facial palsy, and studies that did not report any treatment and/or following outcome for traumatic facial palsies. No minimum study population was required. No publication date restriction was applied.

Abstract and full texts were reviewed in duplicate by different authors. At the abstract review stage, we included all studies that were deemed eligible by at least one rater. At the full-text stage, disagreements were resolved by achieving consensus among raters.

### PICOS criteria

The Population, Intervention, Comparison, Outcomes, and Study (PICOS) framework for the review was defined as follows:

#### P

all patients with traumatic facial palsy.

#### I

any kind of treatment for traumatic facial palsy, either conservative, surgical or combined.

#### C

comparisons between different kinds of treatments and with no treatment.

#### O

recovery after treatment.

#### S

original studies of any kind and clinical setting (except case reports, systematic reviews and meta-analyses).

### Data extraction and quality assessment

For each article included, we recorded: study type, the overall number of included patients, female to male ratio, patients’ age at diagnosis, pre-treatment assessment, deficit degree at presentation before treatment, type of treatment (conservative, surgical or both), adopted parameters of treatment’s selection, post-treatment assessment and results in facial nerve’s recovery after treatment. We excluded papers that did not report post-treatment outcomes or articles in which the selected type of treatment was not specified. Two authors extracted data and rated studies in duplicate, and disagreements were resolved by consensus.

Studies were assessed for both quality and methodological bias according to the National Heart, Lung, and Blood Institute Study Quality Assessment Tools (NHI-SQUAT) [9]. With the same methodology adopted for systematic reviews with middle-to-low evidence levels in comparable recent reviews [10], items were rated as “good” if they fulfilled at least 80% of the items reported in the NHI-SQUAT, “fair” if they fulfilled between 50% and 80% of the items, and “poor” if they fulfilled less than 50% of the items, respectively.

The level of evidence for clinical studies was scored according to the Oxford Centre for Evidence-based Medicine (OCEBM) level of evidence guide [11].

Due to the considerable heterogeneity of study populations, study methods, and the predominantly qualitative nature of collected data, no initial meta-analysis was planned or performed *a posteriori*.

## Results

**Fig. 1:**
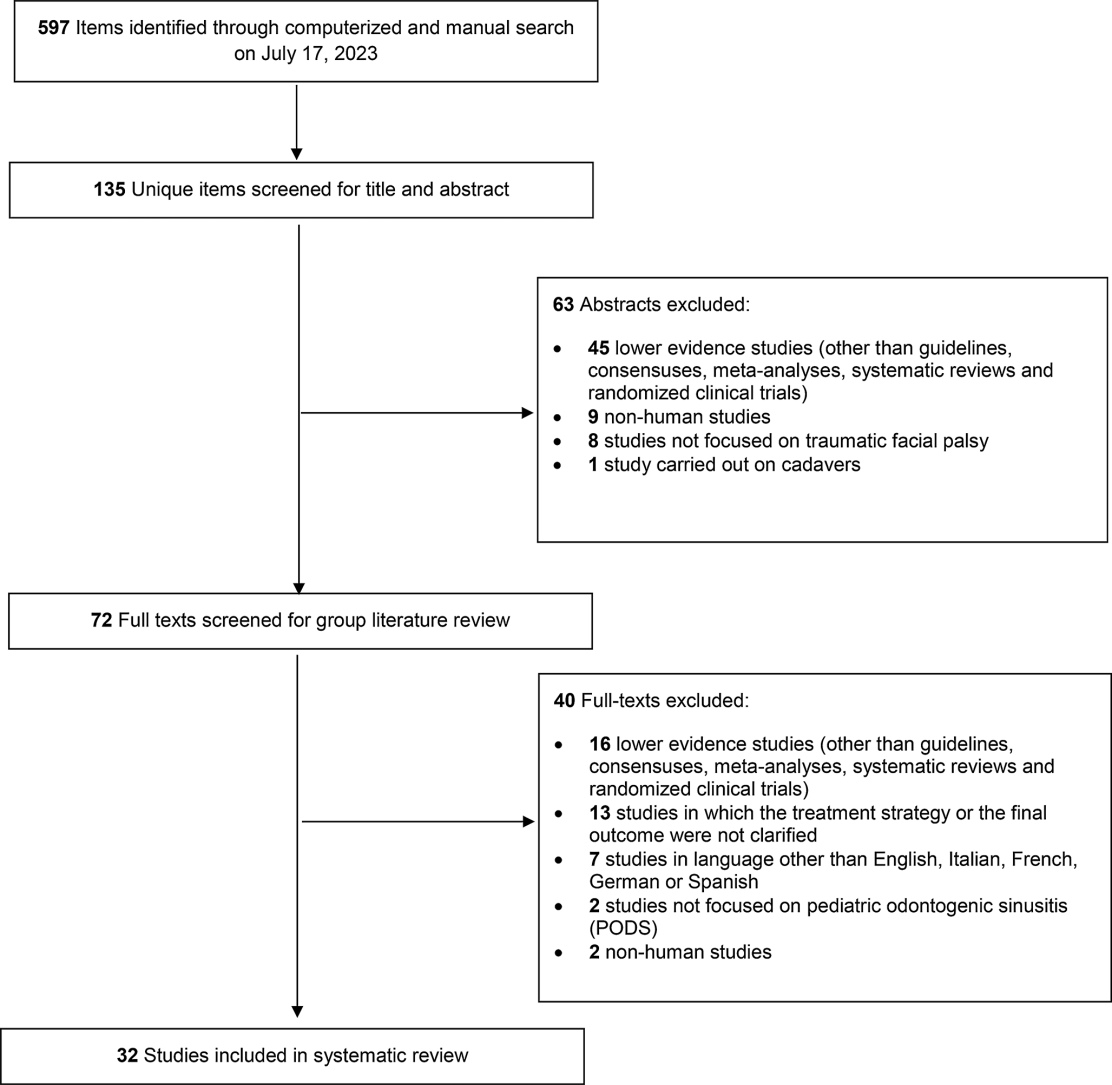
PRISMA style flow diagram of studies through systematic review

Among the 135 unique research items initially identified, 72 published reports were selected for full-text evaluation. No further report was identified from full-text evaluation after reference checking. Overall, 32 studies published between 1957 and 2022 were retained for analysis (Fig. 1) [3–6, 12–39]. The majority (30 out of 32) of reports were case series (level of evidence II according to the OCEBM scale), and only 2 reports were case-control studies (level of evidence IV). Articles were rated as good (*n* = 18), fair (*n* = 11), or poor (*n* = 3) according to NHI-SQAT tool. No significant biases toward the objectives of our review were identified. Table 2 reports the study type, evidence, and quality rating for all studies included.

**Table 2:** Type of study, and evidence and quality rating of reviewed articles

Reference	Study type	OCEBM rating	Quality rating
Adour et al., 1977 [12]	CS	4	G
Al Tawil et al., 2010 [13]	CS	4	G
Alicandri-Ciufelli et al., 2020 [4]	CS	4	G
Aslan et al., 2014 [14]	CS	4	G
Bae et al., 2023 [3]	COS	2	G
Bahadur et al., 1982 [15]	CS	4	F
Baysal et al., 2016 [16]	CS	4	F
Bodenez et al., 2005 [17]	CS	4	G
Briggs et al., 1967 [18]	CS	4	F
Erkan et al., 2022 [19]	CS	4	F
Ferreira et al., 2004 [20]	CS	4	G
Garcia- Fructuos et al., 2000 [21]	CS	4	G
Hai–jin et al., 2011 [22]	CS	4	F
Kacker et al., 1982 [23]	CS	4	P
Kettel, 1958 [24]	CS	4	P
Kettel, 1957 [25]	CS	4	G
Kim et al., 2022 [26]	CS	4	F
Kim et al., 2010 [27]	CS	4	F
Kim et al., 2016 [28]	CS	4	G
Lee et al., 2018 [29]	CS	4	G
Nam et al., 2019 [30]	CS	4	G
Nishant et al., 2018 [31]	CS	4	F
Patnaik et al., 2019 [32]	CS	4	G
Psillas et al., 2007 [33]	CS	4	G
Richards, 1973 [34]	CS	4	P
Shu et al., 2023 [35]	CS	4	G
Ulug et al., 2005 [36]	CS	4	G
Ulug et al., 2009 [37]	CS	4	F
Vajpayee et al., 2018 [5]	CS	4	G
Wamkpah et al., 2022 [38]	COS	2	F
Yadav et al., 2018 [39]	CS	4	G
Yetiser et al., 2008 [6]	CS	4	F

CS, case series; COS, cohort-study; OCEBM, Oxford Centre for Evidence Based Medicine; G, good; F, fair; P, poor

The 32 included studies had 2079 participants whose ages at facial palsy’s presentation ranged from 0 (48-old-newborns) to 70 years (median 24.8 years, interquartile range 16). Patients were more frequently male (female/male ratio 0.45).

**Fig. 2:**
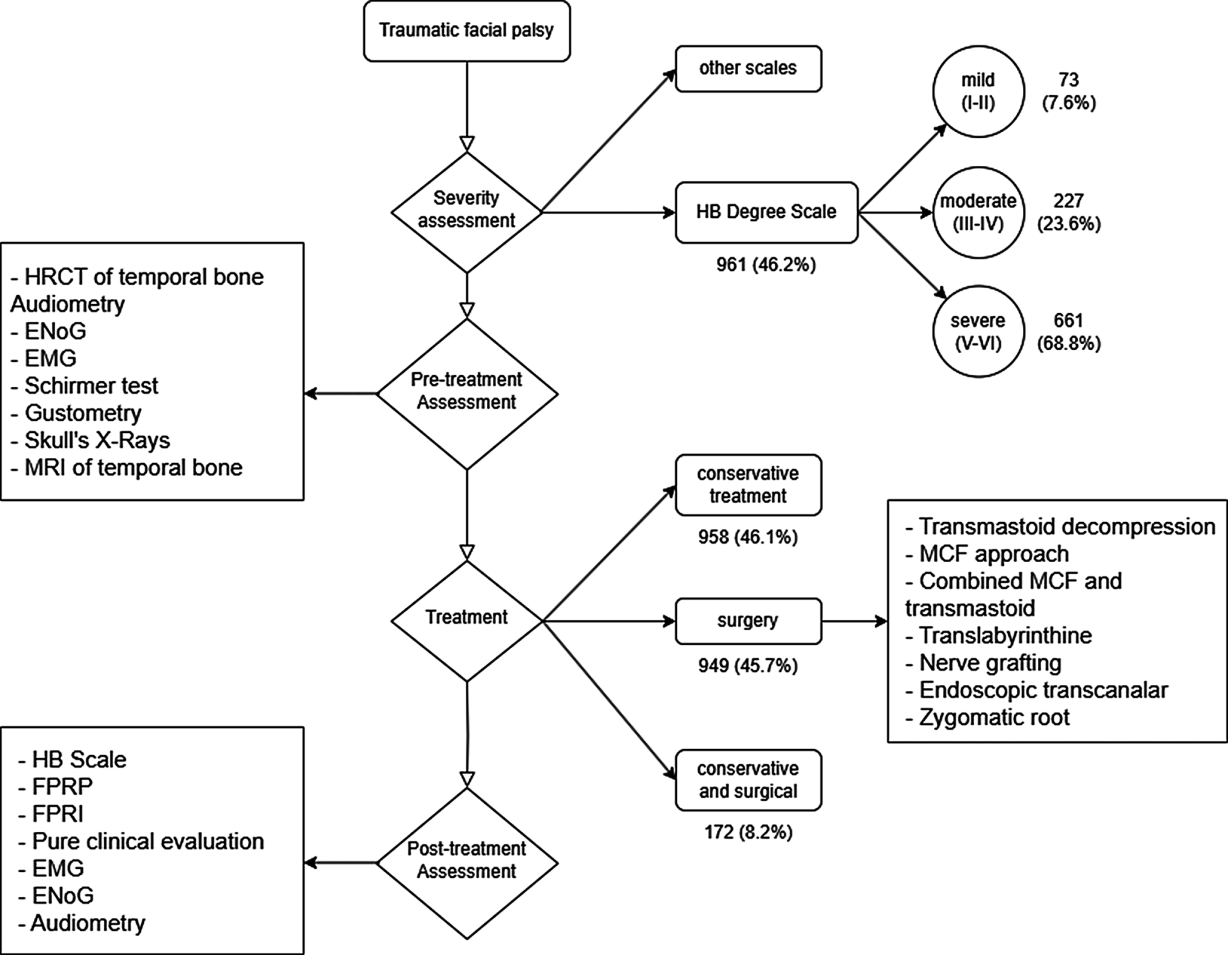
Traumatic facial palsy management. HRCT, High Resolution Computed Tomography, ENOG, electroneurography; EMG, electromyography; MRI, Magnetic Resonance Imaging; MCF, middle cranial fossa; HB, House-Brackmann; FPRP, Facial Paralysis Recovery Profile; FPRI, Facial Paralysis Recovery Index

The flow-chart in Fig. 2 shows the management of traumatic facial palsy derived from this review. In particular, the severity assessment’s method, the types of adopted treatment and the pre- and post-treatment assessments are synthetized.

Table 3 shows the demographic and clinical information for treated patients of every study included in the review, while Table 4 summarize all the results of severity evaluation with House-Brackmann (HB) Degree Scale and all the radiological or clinical tests used for the pre-assessment evaluation. In Table 5 are summarized all the selected treatments for facial palsy and all the available post-treatment tools. All the single data obtained from every study included in the review are shown in Table 6.

**Table 3:** Demographic and clinical information on the treated patients for all included studies

Reference	Treated patients (*n*)	Female: male ratio (*n*: *n*)	Patients’ mean age at diagnosis (years)	Pre-treatment assessment	HB grade
Adour et al., 1977 [12]	30	8:22	30.8	N/R	N/A
Al Tawil et al., 2010 [13]	29	N/R	0 (48–72 h)	Pure clinical data	N/A
Alicandri-Ciufelli et al., 2020 [4]	6	N/R	42.5	HRCT, audiometry	IV, V, VI
Aslan et al., 2014 [14]	13	1:12	30.6	ENoG, EMG, audiometry	IV, VI
Bae et al., 2023 [3]	29	4:29	42.8	ENoG, EMG	VI
Bahadur et al., 1982 [15]	40	14:26	25	Audiometry, Schirmer test, gustometry, X-rays	N/A
Baysal et al., 2016 [16]	15	8:7	20.6	CT, ENoG, EMG	VI
Bodenez et al., 2005 [17]	59	11:48	35	CT, ENoG, EMG, audiometry	N/A
Briggs et al., 1967 [18]	16	N/R	37.5	Radiological exams (not specified)	N/A
Erkan et al., 2022 [19]	13	1:12	34.4	ENoG, EMG	IV, V, VI
Ferreira et al., 2004 [20]	156	67:89	33	HRCT, audiometry, ENoG	IV, V, VI
Garcia- Fructuos et al., 2000 [21]	23	N/R	N/R	HRCT, EMG	N/A
Hai–jin et al., 2011 [22]	33	16:17	33	HRCT, audiometry, Schirmer test, gustometry, ENoG, EMG	III, IV, V
Kacker et al., 1982 [23]	10	N/R	N/R	N/R	N/A
Kettel, 1958 [24]	4	N/R	N/R	N/R	N/A
Kettel, 1957 [25]	188	N/R	N/R	EMG, X-rays	N/A
Kim et al., 2022 [26]	520	88:432	42.4	ENoG	N/A
Kim et al., 2010 [27]	58	N/R	N/R	ENoG, EMG, CT, audiometry	N/A
Kim et al., 2016 [28]	15	7:13	40	CT, MRI, audiometry, ENoG	IV, V, VI
Lee et al., 2018 [29]	26	10:16	37.2	CT, ENoG, EMG, audiometry	III, IV, V, VI
Nam et al., 2019 [30]	12	21:28	43.3	CT, ENoG, EMG, audiometry	N/R
Nishant et al., 2018 [31]	40	N/R	N/R	CT, audiometry	N/R
Patnaik et al., 2019 [32]	11	4:7	41.2	CT, ENoG, EMG, audiometry	V, VI
Psillas et al., 2007 [33]	350	164:156	42.4	ENoG	II, III, IV, V, VI
Richards, 1973 [34]	4	2:2	N/R	X-rays, ENoG, audiometry	N/R
Shu et al., 2023 [35]	11	5:6	39	ENoG, CT, audiometry	VI
Ulug et al., 2005 [36]	10	3:7	23.6	ENoG, CT, EMG, audiometry	N/A
Ulug et al., 2009 [37]	3	2:1	5	ENoG, EMG, CT, audiometry	N/A
Vajpayee et al., 2018 [5]	28	5:23	24	CT, audiometry	II, III, IV, V, VI
Wamkpah et al., 2022 [38]	263	214:524	32.6	CT, EMG	N/A
Yadav et al., 2018 [39]	39	5:34	33.5	ENoG, HRCT, Schirmer test	II, III, IV, V, VI
Yetiser et al., 2008 [6]	25	5:30	24.1	ENoG, HRCT, Schirmer test	IV, V, VI

N/R, not reported; N/A, not adopted; HB, House-Brackmann; HRCT, high-resolution computed tomography; ENoG, electroneuronography; EMG, electromyography; CT, computed tomography; MRI, magnetic resonance imaging

**Table 4:** Severity evaluation results (HB scale) and pre-treatment assessment tools

HB Degree Scale → 18/32 (56.3%) studies, 961/2079 (46.2%) patients	
Degree (severity)	Cases (%)
I-II (mild)	73 (7.6)
III-IV (moderate)	227 (23.6)
V-VI (severe)	661 (68.8)

Pre-treatment assessment → 4031 assessments	
Type of assessment	Cases (%)
Temporal HRCT/CT	818 (20.3)
Audiometry	525 (13.0)
ENoG	1402 (34.8)
EMG	756 (18.8)
Schirmer test	137 (3.4)
Gustometry	73 (2.5)
X-rays	232 (5.8)
Temporal MRI	15 (0.4)
Pure clinical data	29 (0.7)
N/R	44 (1.1)

N/R, not reported; N/A, not adopted; HB, House-Brackmann; HRCT, high-resolution computed tomography; ENoG, electroneuronography; EMG, electromyography; CT, computed tomography; MRI, magnetic resonance imaging

**Table 5:** Treatments and post-treatment assessment tools

Treatment	
Type of treatment	Cases (%)
Surgery	949 (45.7)
Conservative therapy (“wait and see”)	958 (46.1)
Both surgical and conservative therapy	172 (8.2)

Surgical strategies → 949 (45.7) patients	
Type of surgery	Cases *
Transmastoid decompression	63
MCF	178
Combined MCF and transmastoid	9
Translabyrinthine	6
Nerve grafting	12
Endoscopic transcanalar	17
Zygomatic root	3

Post-treatment assessment → 3300 assessments	
Type of assessment	Cases (%)
FPRP	30 (0.9)
FPRI	30 (0.9)
HB scale	1495 (45.3)
Clinical evaluation	262 (7.9)
EMG	354 (10.7)
ENoG	654 (19.8)
Audiometry	162 (4.9)
Endoscopy	11 (0.3)
Otoscopy	10 (0.3)
N/R	292 (8.8)

* in most cases, the type of reported surgical treatment is missing

S, surgery; C, conservative; CR, compete recovery; LR, lack of recovery; PR, partial recovery; FPRP, Facial Paralysis Recovery Profile; FPRI, Facial Paralysis Recovery Index; N/R, not reported; HRCT, high-resolution computed tomography; HB, House-Brackmann; ENoG, electroneuronography; MCF, middle cranial fossa; CT, computed tomography

**Table 6:** Parameters of selected treatment, treatment regimens, outcome, post-treatment assessment

Reference	Treated patients (*n*)	Parameters of selected treatment	Treatment (S/C/both)	Type of surgery	Outcome (*n*)	Post-treatment assessment
Adour et al., 1977 [12]	30	Complete/incomplete facial palsy	S (15), C (15)	Decompression (N/R approach)	CR (10, C); PR (20)	FPRP; FPRI
Al Tawil et al., 2010 [13]	29	N/R	C	-	CR (26), PR (1), N/R (2)	N/R
Alicandri-Ciufelli et al., 2020 [4]	6	Imaging (HRCT)	both	Endoscopic transcanalar	CR (5); PR (1)	HB scale
Aslan et al., 2014 [14]	13	HB grade	S	N/R	PR	HB scale
Bae et al., 2023 [3]	29	HB grade, ENoG	S (21), C (8)	Transmastoid (21), combined transmastoid-MCF (4)	CR (N/R), PR (N/R)	HB scale
Bahadur et al., 1982 [15]	40	N/R	S	Decompression (N/R approach)	CR (2), PR (38)	Clinical evaluation
Baysal et al., 2016 [16]	15	Timing of palsy onset	S	Transmastoid	PR	HB scale, EMG
Bodenez et al., 2005 [17]	59	Complete/incomplete facial palsy	S (26), C (33), both (5)	Decompression (N/R approach) (40), mixed (14), translabyrinthine (5)	CR (24, C; 10, S), PR (25)	HB scale, EMG
Briggs et al., 1967 [18]	16	Imaging (HRCT)	C	-	CR (14); PR (2)	Clinical evaluation
Erkan et al., 2022 [19]	13	ENoG	S	MCF	PR	HB scale
Ferreira et al., 2004 [20]	156	Imaging, ENoG	both	MCF	CR (68); PR (87); LR (1)	HB scale
Garcia- Fructuos et al., 2000 [21]	23	Timing of palsy onset, EMG	S (2), C (21)	Decompression (N/R approach)	CR (N/R), PR (N/R), died (1)	HB scale
Hai–jin et al., 2011 [22]	33	Imaging (HRCT), ENoG	S	Transmastoid	CR (N/R), PR (N/R)	HB scale
Kacker et al., 1982 [23]	10	N/R	S	Nerve grafting	PR (9), LR (1)	Clinical evaluation
Kettel, 1958 [24]	4	N/R	S	Decompression (N/R approach)	PR	Clinical evaluation
Kettel, 1957 [25]	188	Timing of palsy onset, EMG	S	Decompression (N/R approach) (N/R), nerve grafting (N/R)	CR (N/R), PR (N/R), LR (35)	Clinical evaluation, EMG
Kim et al., 2022 [26]	520	N/R	S (410), C (110)	Trasmastoid (N/R), translabyrinthine (N/R), MCF (N/R)	CR (N/R), PR (N/R); better outcomes in the surgical group	HB scale, ENoG
Kim et al., 2010 [27]	58	CT	S (52), C (6)	MCF (N/R), transmastoid (N/R)	PR	HB scale, ENoG, audiometry
Kim et al., 2016 [28]	15	N/R	S	Transmastoid	CR (9), PR (6)	HB scale, audiometry
Lee et al., 2018 [29]	26	N/R	C	-	CR (N/R), PR (N/R)	HB scale, ENoG
Nam et al., 2019 [30]	12	Comorbidities	C (high/moderate/low steroid)	-	CR (N/R), PR (N/R)	HB scale
Nishant et al., 2018 [31]	40	N/R	S (6), C (34)	Transmastoid (4), MCF (2)	CR (28), PR (6), LR (6)	HB scale
Patnaik et al., 2019 [32]	11	ENoG	S	Transmastoid	CR (2), PR (9)	HB scale
Psillas et al., 2007 [33]	350	N/R	C	-	CR (N/R), PR (N/R)	HB scale
Richards, 1973 [34]	4	X-rays	S (2), C (2)	Transmastoid	CR	Clinical evaluation, audiometry
Shu et al., 2023 [35]	11	Failure of conservative treatment	S	Endoscopic transcanalar	CR (N/R), PR (N/R)	HB scale, audiometry, endoscopy, ENoG
Ulug et al., 2005 [36]	10	CT, ENoG	S	MCF (7), combined MCF and transmastoid (3)	PR	HB scale, audiometry, otoscopy
Ulug et al., 2009 [37]	3	CT	S	Zygomatic root approach	PR (2), N/R (1)	HB scale
Vajpayee et al., 2018 [5]	28	Timing of palsy onset	S (10), C (18)	Transmastoid	CR (N/R), PR (N/R)	HB scale, EMG
Wamkpah et al., 2022 [38]	263	EMG	S (8), C	Decompression (N/R approach)	CR (N/R), PR (N/R)	N/R
Yadav et al., 2018 [39]	39	N/R	S (7), C (32)	Decompression (N/R approach)	CR (N/R), PR (N/R)	HB scale, EMG, ENoG, audiometry
Yetiser et al., 2008 [6]	25	N/R	S (13), C (1), both (11)	Combined MCF and transmastoid (2), translabyrinthine (1), transmastoid (13), nerve grafting (2)	CR (3), PR (22)	HB scale, EMG, audiometry

S, surgery; C, conservative; CR, compete recovery; LR, lack of recovery; PR, partial recovery; FPRP, Facial Paralysis Recovery Profile; FPRI, Facial Paralysis Recovery Index; N/R, not reported; HRCT, high-resolution computed tomography; HB, House-Brackmann; ENoG, electroneuronography; MCF, middle cranial fossa; CT, computed tomography

The parameters for selecting the appropriate treatment were also analyzed: 2 studies considered the type of paralysis (complete versus incomplete), 4 studies considered the timing of the palsy’s onset after the temporal trauma, and 2 studies considered the severity based on the HB grade. Imaging (HRCT, CT, X-rays) was used as a pre-assessment tool to select the most appropriate treatment in 7 studies, while 9 studies based their therapeutic decisions using ENoG or EMG. Lastly, 1 study based the therapeutic strategy decision on the lack of response to corticosteroid therapy [35], and 1 study considered patients’ comorbidities to administer high, medium or low doses of steroids to treat the palsy [30]. In 11 studies, the parameters for selecting the therapeutic strategy were not reported.

As regards the outcome of treatments, we considered:

- Complete recovery (CR): grade I-II on the HB Scale or a Recovery Profile of + 10 on the Facial Paralysis Recovery Profile (FPRP) at the end of the follow-up.
- Partial recovery (PR): grade III-IV-V on the HB Scale or a Recovery Profile between + 3 and + 9 on the FPRP at the end of follow-up.
- Lack of recovery (LR): grade V-VI on the HB Scale or a Recovery Profile less than + 3 on the FPRP at the end of follow-up.

Outcomes of selected treatments are collected in Table 6, and where available, the proportions of patients who experienced complete, partial, or lack of recovery with conservative, surgical, or both treatments are reported. In general, we assume the following points:

- Conservative therapy (17 out of 32 studies): CR obtained in 16 studies, PR in 18 studies, LR in 1 study.
- Surgical therapy (25 out of 32 studies): CR obtained in 16 studies, PR in 24 studies, LR in 3 studies.
- Both conservative and surgical therapy (4 out of 32 studies): CR was obtained in 4 studies, PR in 4 studies, LR in 1 study.

The extreme heterogeneity of patient selection methods and outcome indicators has made any meta-analytic work comparing the various treatments impossible.

## Discussion

In this systematic review, we aimed to determine the best therapeutic strategy for traumatic facial palsy. Analyzing the existing literature on this topic, we found only a few eligible studies with a low level of evidence (30 out of 32 are case series); this demonstrates the huge variability of this theme and the lack of standardized guidelines for therapeutic strategies for this condition. This is attributable to the extreme heterogeneity of available pre-treatment diagnostic tools (in total, 8 different tools were used among all the papers of this review, as reported in Table 3) and of available therapeutic strategies (due mostly to the huge number of existing surgical approaches for facial palsy).

Moreover, an important bias lies in the treatment selection: in most of the papers, the worst palsies were immediately treated with decompression without waiting, especially if the trauma had led to other complications, while clinical management alone was mostly considered for late-onset or incomplete palsies. This makes it difficult to standardize the definition of the best treatment choice. Furthermore, the fact that many studies report mixed treatments increases the difficulty in establishing which treatment has the best outcome. Some studies consider surgical treatment after failure of medical one [5], while others propose concomitant medical treatment during surgery recovery [20]. In the study by Nam et al., medical therapy was administered to all the patients in different doses and in different durations based on the decision of distinct professional otorhinolaryngologists. The high dose consisted of methylprednisolone 634.7 mg for 12 days, the moderate dose was 496 mg for 12 days, and the low dose was 344 mg for 10 days. It turned out that the degree of recovery was not significantly different among the groups, indicating no superiority of one protocol over the others [30].

As for the considered follow-up period, different periods of post-treatment recovery are reported: some studies describe the outcome considering just the first month after the treatment, others extend it to 3 months [17, 30], while others follow patients’ recovery for up to 1 year after the treatment [3, 29]. By doing so, there is no way to establish the superiority of one specific treatment over another within an accurate and defined follow-up time. Additionally, the post-treatment evaluation differs from one study to another, although the most used assessment was the HB scale. This heterogeneity does not facilitate the distinction between a complete recovery and an incomplete one.

A deeper exploration of the biases and limitations in the current review can be achieved by evaluating the implications of findings for specific subgroups, such as those defined by age and gender. The demographic data from the included studies indicate that patients with traumatic facial palsy are predominantly male (female/male ratio: 0.45), with a median age of 24.8 years and an interquartile range of 16 years. This distribution may reflect underlying differences in the mechanisms of trauma, such as higher exposure to high-energy injuries in young males, or disparities in healthcare-seeking behaviour across genders.

Further stratification of treatment outcomes by these subgroups could provide additional insights. For instance, younger patients might have a higher regenerative capacity, potentially leading to better recovery outcomes with conservative treatment. Conversely, older patients or those with comorbidities might benefit more from surgical interventions, as their recovery potential may be limited by systemic factors. However, no studies in this review explicitly analyzed outcomes based on age, gender, or other demographic factors, highlighting a critical gap in the literature.

The heterogeneity of therapeutic approaches also complicates subgroup analysis. For example, surgical decompression was predominantly used in cases of severe paralysis (House-Brackmann V-VI), often associated with high-energy trauma, while conservative management was favored for incomplete or delayed-onset palsies. It remains unclear whether these treatment patterns align with the specific needs of different subgroups, as the rationale for treatment selection often lacked standardization.

Moreover, the follow-up periods varied significantly across studies, ranging from one month to over a year. This variability could obscure potential differences in recovery trajectories among subgroups. For instance, younger patients might show earlier signs of recovery, while older individuals could exhibit delayed but steady improvement. Additionally, the tools used for post-treatment assessment, such as the House-Brackmann scale, were not uniformly applied, further complicating comparisons across demographic groups.

To address these gaps, future research should prioritize the stratification of patients by demographic factors and incorporate standardized assessment tools and follow-up periods. By doing so, it would be possible to develop tailored treatment protocols that consider the unique needs and recovery potentials of specific subgroups, ultimately improving patient outcomes and reducing disparities in care.

Among all the above-mentioned weak points, a strong one is the large number of patient data analyzed in this systematic review and the fact that there are a few reviews about this topic: this explains the reason why there are no shared guidelines about the management of traumatic facial palsy yet. Through this review, we can confirm that traumatic facial palsy remains a challenge for the ENT (ears, nose, and throat) surgeon, not only because it mostly affects young and healthy people (as we can infer from the patients’ median age at the time of palsy onset, reported in Table 3), but also because the choice of the right treatment is guided by the individual patients’ situation. This includes the type and severity of the trauma, the onset and degree of palsy, the grade of denervation, and the radiologic evidence of facial canal damage.

Even though there is still no way to definitively determine whether surgical decompression, immediate or delayed, leads to a better outcome than prolonged medical treatment, we can establish some key points of action. Above all, good patient selection in the preoperative stage is mandatory: this is essential not only to provide tailored support according to the severity of the palsy, but also to avoid overtreatment or undertreatment. For example, if the palsy is complete and immediate, surgery (of any type according to the extent of facial damage) leads to a good outcome in most of the evaluated studies. Conversely, if the palsy is not complete and/or is delayed, medical treatment is still effective and can lead to satisfactory recovery results.

Above all, we must consider that a surgical option could always be followed by intraoperative and postoperative complications, especially because the facial nerve follows a mixed course, both intracranial and extracranial. Therefore, this type of surgery must be performed in well-established experience centers, where both ENT surgeons and neurosurgeons are available. On the other hand, simple medical treatment and a “wait and see” approach have, of course, fewer complications than a surgical procedure. Thus, the surgeon must consider the cost-benefit balance when making a treatment choice.

In conclusion, we can assume that in the future, more prospective studies should be conducted to define a flowchart for dealing with traumatic facial nerve palsy. To achieve this, we need to carefully stratify patients according to universally shared pre-treatment assessments and post-treatment outcomes. Currently, there is no consensus on the best way to determine if a patient needs surgical treatment due to the extreme heterogeneity of available therapeutic choices and the lack of standardized patients’ stratification.

## Data Availability

All data pertaining to this systematic review are available from the corresponding author upon reasonable request.
